# Supplementary material for: Axin2/Conductin Is Required for Normal Haematopoiesis and T Lymphopoiesis
Source: Cells. 2022 Aug 28;11(17):2679. doi: 10.3390/cells11172679 (PMC9454631; doi:10.3390/cells11172679)
Supplement: Supplementary file 1 [file cells-11-02679-s001.zip › cells-1807632-supplementary.pdf]

Supplementary Materials

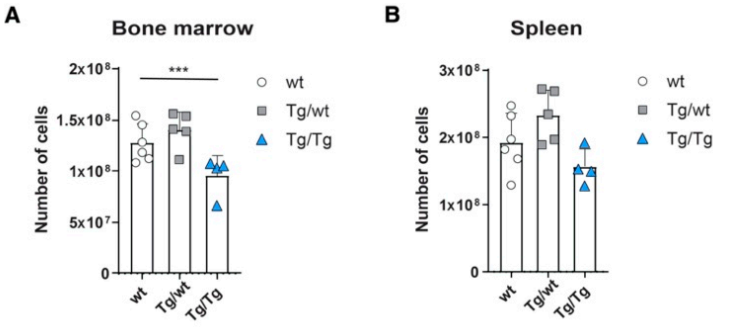

Figure S1: Axin2 deficiency affects slightly total cell numbers in hematopoietic organs. Total cell numbers from bone marrow (**A**) and spleen (**B**) obtained from wild type (wt), heterozygous (Tg/wt) and homozygous (Tg/Tg) Axin2-mTurquoise2 steady state mice. Data represent results from five wild type control mice (wt), five heterozygous Axin2-mTurquoise2 mice (Tg/wt) and four homozygous Axin2-mTurquoise2 mice (Tg/Tg), from two independent experiments. Error bars represent (SEM). ***p < 0.001 (ANOVA test).

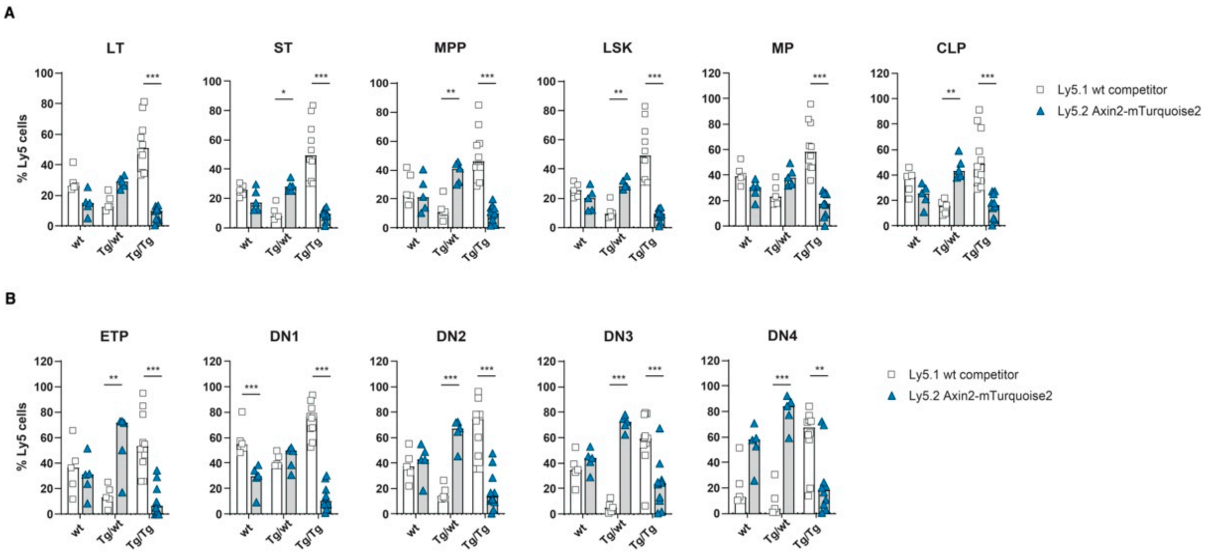

Figure S2: Repopulation efficiency analyzed in bone marrow (**A**) and thymus (**B**) of mice transplanted with Ly5.2 wilde type (wt), heterozygous Axin2-mTurquoise2 (Tg/wt) and homozygous Axin2-mTurquoise2 (Tg/Tg) and Ly5.1 wild type control HSCs, 6 weeks after transplantation. Data represent results from five wild type control mice (wt), five heterozygous Axin2-mTurquoise2 mice (Tg/wt) and ten homozygous Axin2-mTurquoise2 mice (Tg/Tg), from two independent experiments. Error bars represent (SEM). *p 0,033, **p 0,002 and ***p < 0.001 (ANOVA test).
